# Connexin43 represents an important regulator for Sertoli cell morphology, Sertoli cell nuclear ultrastructure, and Sertoli cell maturation

**DOI:** 10.1038/s41598-022-16919-4

**Published:** 2022-07-28

**Authors:** Sarah Staggenborg, Rüdiger Koch, Kristina Rode, Hanna Hüneke, Louiza Tiedje, Gudrun Wirth, Marion Langeheine, Ines Blume, Kerstin Rohn, Christoph Wrede, Christiane Pfarrer, Ralph Brehm

**Affiliations:** 1 Institute of Anatomy, University of Veterinary Medicine Hannover, Foundation, Bischofsholer Damm 15, 30173 Hannover, Germany; 2 Institute of Pathology, University of Veterinary Medicine Hannover, Foundation, Hannover, Germany; 3 Institute of Functional and Applied Anatomy, Research Core Unit Electron Microscopy, Hannover Medical School, Hannover, Germany

**Keywords:** Nucleus, Nuclear organization, Differentiation, Cells

## Abstract

The Sertoli cell (SC)-specific knockout (KO) of connexin43 (Cx43) was shown to be an effector of multiple histological changes in tubular morphology, resulting in germ cell loss through to a Sertoli-cell-only (SCO) phenotype and vacuolated seminiferous tubules containing SC-clusters. Our present study focused on the effects of Cx43 loss on SC ultrastructure. Using serial block-face scanning electron microscopy (SBF-SEM), we could confirm previous results. Ultrastructural analysis of Sertoli cell nuclei (SCN) revealed that these appear in clusters with a phenotype resembling immature/proliferating SCs in KO mice. Surprisingly, SCs of fertile wild type (WT) mice contained SCN with a predominantly smooth surface instead of deep indentations of the nuclear envelope, suggesting that these indentations do not correlate with germ cell support or spermatogenesis. SBF-SEM facilitated the precise examination of clustered SCs. Even if the exact maturation state of mutant SCs remained unclear, our study could detect indications of cellular senescence as well as immaturity, emphasising that Cx43 affects SC maturation. Moreover, Sudan III staining and transmission electron microscopy (TEM) demonstrated an altered lipid metabolism in SCs of Cx43 deficient mice.

## Introduction

Sertoli cells (SC) occupy approximately 15% of the adult murine seminiferous epithelium^1,2^. They are columnar shaped, extending from the basement membrane of the seminiferous epithelium with long cytoplasmic branches towards the tubular lumen^2^. The large, pyramidal shaped Sertoli cell nucleus (SCN) resides near the tubular basement membrane in adult mice^3^, showing deep indentations of the nuclear envelope. Little is known about the function of these nuclear infoldings. So far, in those indentations vimentin filaments have been detected^4^. Moreover, microtubules and f-actin could also be found in these regions, but studies have shown that their presence is dispensable for the maintenance of SC nuclear shape^5^. The large, euchromatic nucleolus is tripartite with mainly two satellite chromocenters, in which most of the SC’s heterochromatin is organized. This unique morphology constitutes a special feature of the mature SC^6^. The nuclear shape changes in the developing testis from round to oval in neonatal mice, to elongated during puberty through to polygonal with deep indentations of the nuclear envelope^7^.

Gap junctions enable direct cellular communication^8^. A gap junction channel consists of two hemichannels, which are composed of six protein subunits, called connexins^8,9^. Connexin43 (Cx43) represents the predominant gap junction protein within the testis^10,11^ and occurs within the seminiferous epithelium mostly between adjacent SCs, between SCs and spermatogonia and primary spermatocytes^12^. Moreover, it is known to be a pubertal SC differentiation/maturation marker^11,13^.

Mammalian SCs are presumed to be terminally differentiated cells like neurons. In rodents, their proliferation ceases during puberty between two and three weeks of age^14–17^. However, it was proved that SCs are still proliferating in adult mice with a homozygous SC-specific knockout of Cx43 (SCCx43KO^−/−^), indicating that Cx43 plays a key role in the regulation of SC proliferation and maturation^18–20^. Moreover, structural alterations of seminiferous tubules occur in the seminiferous epithelium of homozygous adult mutant mice. Tubules display germ cell loss, resulting in the arrest of spermatogenesis at the level of spermatogonia or Sertoli-cell-only (SCO) syndrome. These seminiferous tubules are smaller in diameter compared to those of their WT littermates and are often vacuolated, with intratubular located, clustered SCs^18,20–22^. Furthermore, it was found that SC numbers per seminiferous tubule are increased in SCCx43KO^−/−^ mice^18,21^. Although the tubular structure is altered, it was verified that the integrity of the blood-testis-barrier persists in mutants^23–25^.

Rat SCs have been reconstructed in three-dimensional level for the first time by Wong and colleagues using time consuming semi-serial thin sectioning techniques and serial reconstruction techniques^26^. Serial block-face scanning electron microscopy (SBF-SEM) is a relatively new technique to process resin-embedded tissue samples. The method offers various advantages compared to conventional transmission electron microscopy (TEM): the slicing and imaging process runs completely automatic, which makes it time-saving for the examiner^27^ and creates homogenous images. Together with a large field of view this allows imaging of specimen blocks of a greater volume than with TEM techniques^28^. Since the slice thickness is only a few nanometres, the Z-axis of tissues can be imaged without almost any loss of information, thus specimens could be examined with greater ease not only two-dimensionally, but also in three-dimensional level.

## Results

### Sudan III staining

**Figure 1:**
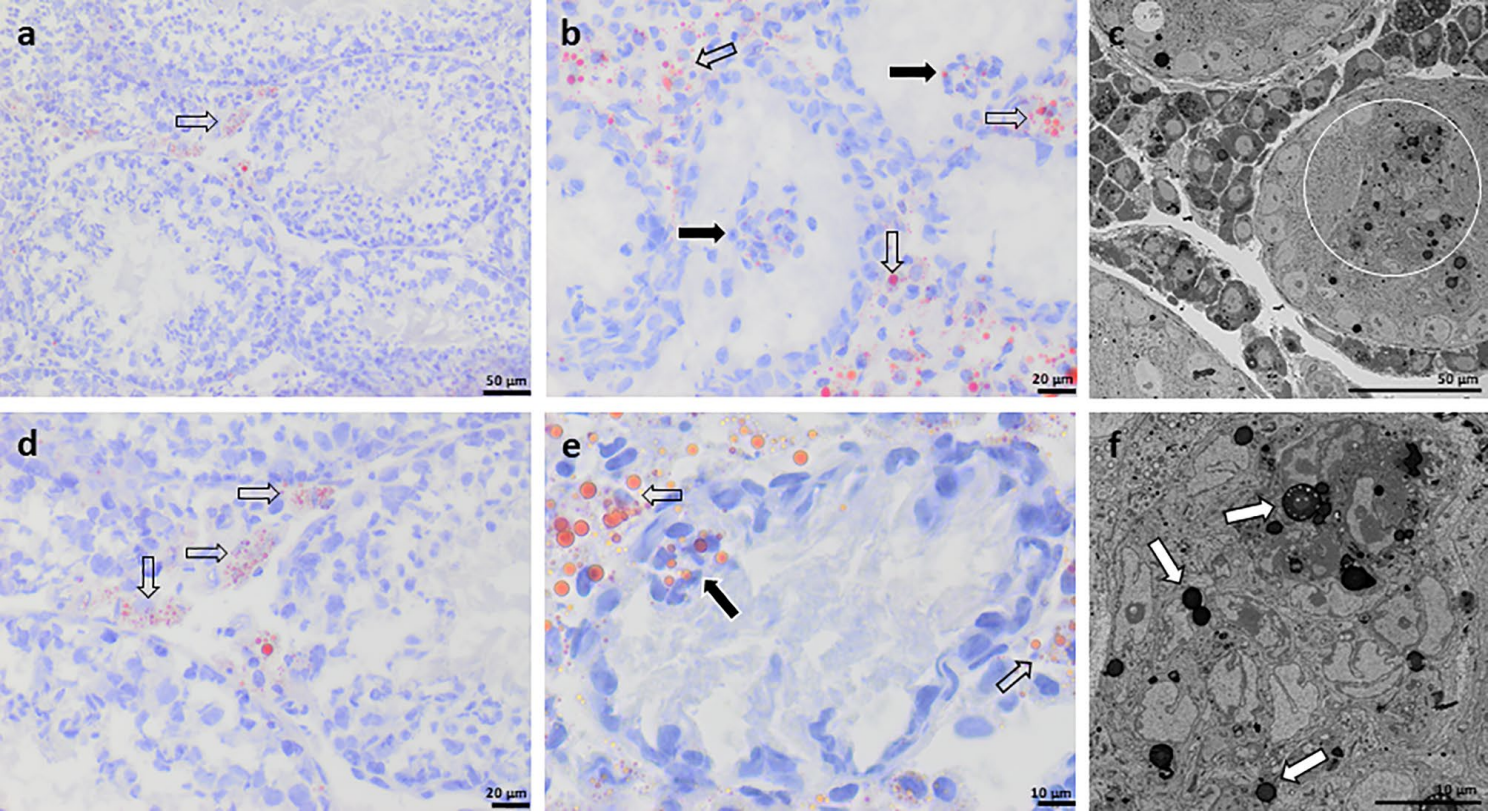
Sudan III staining of murine wild type (WT) (**a**,**d**) and SCCx43KO^−/−^ (**b**,**e**) testes. In WT testes Sudan III-stained granules particularly occur in the cytoplasm of interstitial Leydig cells (LC) (transparent arrows) and rarely in Sertoli cells (SC). A similar distribution pattern is seen in LCs of SCCx43KO^−/−^ mice (transparent arrows), but granules are larger and more numerous. Especially SCs of intratubular SC-clusters also contain abundant Sudan III-stained granules (black arrows). Serial block-face scanning electron microscopy sections of a SCCx43KO^−/−^ testis (**c**,**f**). Lipid droplets are concentrated in the tubular SC-clusters (**c**, white circle). Higher magnification displays that lipids are accumulated in the SC’s cytoplasm (**f**, white arrows). Scale bars (**a**,**c**) = 50 µm, (**b**,**d**) = 20 µm, (**e**,**f**) = 10 µm.

In testes of wild type (WT) mice, intracytoplasmic Sudan III-stained granules occurred particularly in interstitial Leydig cells (LC) and were rarely found in SCs (Fig. 1a,d). A similar interstitial distribution pattern was also seen in testes of SCCx43KO^−/−^ mice, but granules were distinctly larger and more abundant. While SCs of WT mice showed only faint Sudan III staining, intratubular SC-clusters in mutants contained SCs with numerous lipid droplets (Fig. 1b,e). Intratubular vacuoles did not contain Sudan III-stained material. SBF-SEM could confirm the distribution pattern of lipid droplets in intratubular cell-clusters of SCCx43KO^−/−^ mice (Fig. 1c,f, Supplementary Video S1).

Counting of lipid droplets in tubule cross-sections by light microscopy resulted in a 5.764-fold higher average number in the 68 days old mutant compared to his WT littermate. This difference was even stronger in the 138 days old SCCx43KO^−/−^ male, who showed a 12.59-fold increase. The evaluation of seminiferous tubules using ImageJ could confirm these results: at the age of 68 days, the number of lipid droplets per tubule cross-section was 5.698-fold higher, at the age of 138 days the increase was 8.675-fold. In addition, using TEM it could be shown that the average number of lipid droplets in SCs at the basal compartment of the seminiferous tubule in mutant mice is 3.294-fold higher per field of view. Moreover, having a closer look on lipid droplets using TEM revealed that these lipid droplets are often arranged in small groups in SCs of mutants (Supplementary Fig. S1c,e,f). These groups did also rarely occur in SCs of WT mice, but seemed to contain less droplets (Supplementary Fig. S1b).

### Effects of loss of Sertoli cell-Cx43 on Sertoli cell nuclear ultrastructure

**Figure 2:**
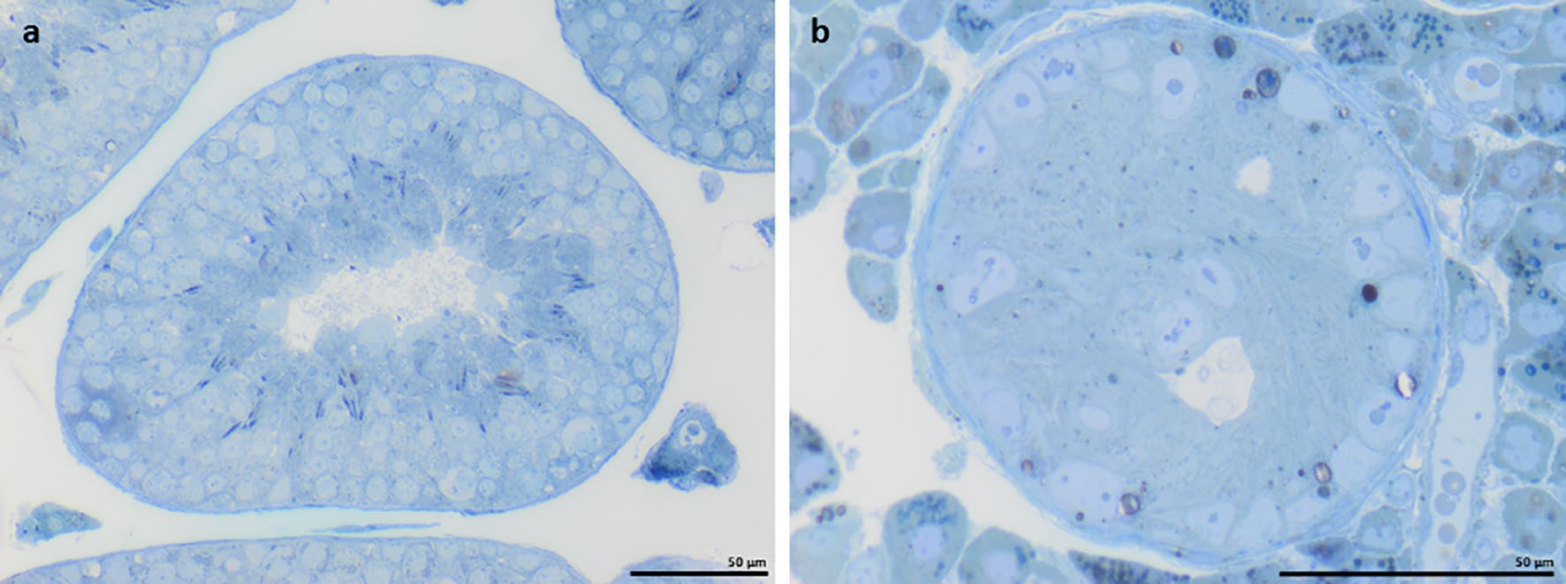
Toluidine blue-stained semithin sections. Seminiferous tubules of wild type (**a**) and SCCx43KO^−/−^ (**b**) mice. Seminiferous tubules of SCCx43KO^−/−^ mice are noticeably smaller in diameter and vacuolated, with intratubular Sertoli cell-clusters. Scale bars = 50 µm.

**Figure 3:**
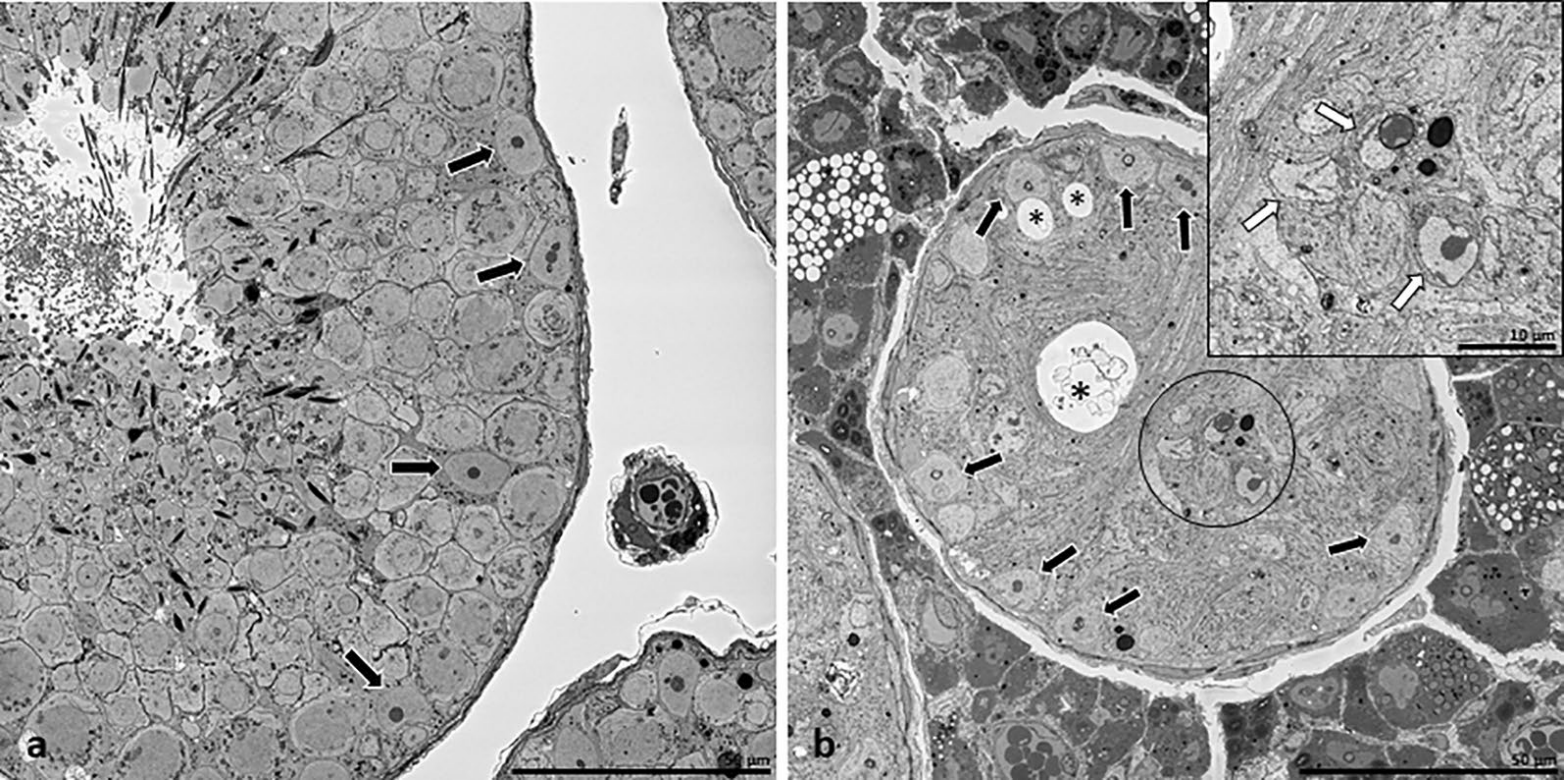
Traverse serial block-face scanning electron microscopy sections of seminiferous tubules of wild type (WT) (**a**) and SCCx43KO^−/−^ (**b**) mice. All germ cell stages are detectable in seminiferous tubules of WT mice (**a**). Note few Sertoli cell nuclei (SCN) near the basal lamina (black arrows). Seminiferous tubules of mutants (**b**) are smaller in diameter and vacuolated (black asterisks). SCN can be found near the basal lamina (black arrows) as well as a part of intratubular Sertoli cell (SC)-clusters (black circle). Scale bars = 50 µm. SCN of SC-clusters (inset, white arrows) show characteristics of immaturity: a small diameter and a polygonal shape with heterochromatin patches along the nuclear membrane. These SCs do not have the typical shape, with long branches reaching the tubular lumen, but have a small cytoplasmatic edge, showing no signs of cellular polarity. Scale bar = 10 µm.

**Figure 4:**
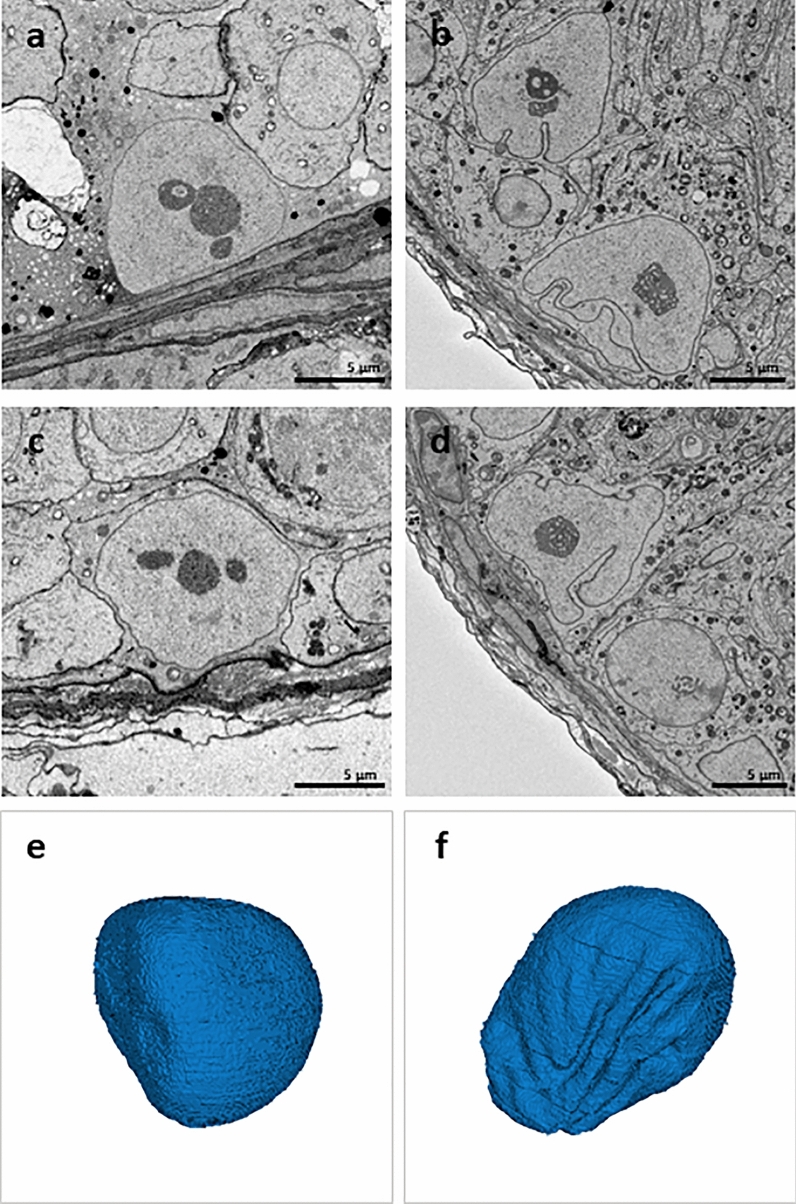
Sertoli cell nuclei (SCN) of wild type (WT) (**a**,**c**) and SCCx43KO^−/−^ (**b**,**d**) mice imaged using serial block-face scanning electron microscopy. 3D rendering of Sertoli cell nuclei (SCN) of WT (**e**) and SCCx43KO^−/−^ (**f**) mice. SCN of WT mice have a round to oval shape with little and shallow indentations of the nuclear envelope (**a**,**c**,**e**). The seminiferous epithelium of SCCx43KO^−/−^ mice contains SCN with deep and numerous indentations (**b**,**d,f**). The characteristic tripartite nucleolus occurs in SCN of both genotypes. Scale bars = 5 µm.

Loss of Cx43 in SCs had different effects on tubular structure and morphology (Figs. 2, 3, Supplementary Fig. S2, Supplementary Video S2). In addition, SCN of WT mice showed a typical mature phenotype, but, surprisingly, less indentations of the nuclear envelope as expected from literature. SCN mainly had a round to oval shape (Fig. 4a,c,e) and were located near the tubular basement membrane (Fig. 3a). They contained mostly two satellite chromocenters, less common ones, forming the characteristic tripartite nucleolus. No heterochromatin patches could be found near the nuclear membrane. In contrast, seminiferous tubules of SCCx43KO^−/−^ mice contained two types of SCN: basally located SCN with a mature phenotype and SCN of SC-clusters (Fig. 3b, Supplementary Video S1) showing signs of immaturity. SCN of mutants were numerously and deeply intended (Fig. 4b,d,f, Supplementary Video S3). In basally located SCN, nuclear clefts could be found particularly at the basal and apical surface, whereas in clustered SCN, clefts were diffusely distributed over the nuclear surface. In SCCx43KO^−/−^ mice, the most obvious alterations occurred in SCN of intratubular SC-clusters and could be seen as features of immaturity: these SCN had a smaller diameter and a polygonal shape, plus heterochromatin patches along the nuclear envelope (Fig. 3, insert, Supplementary Video S1).

### Special nuclear morphology

**Figure 5:**
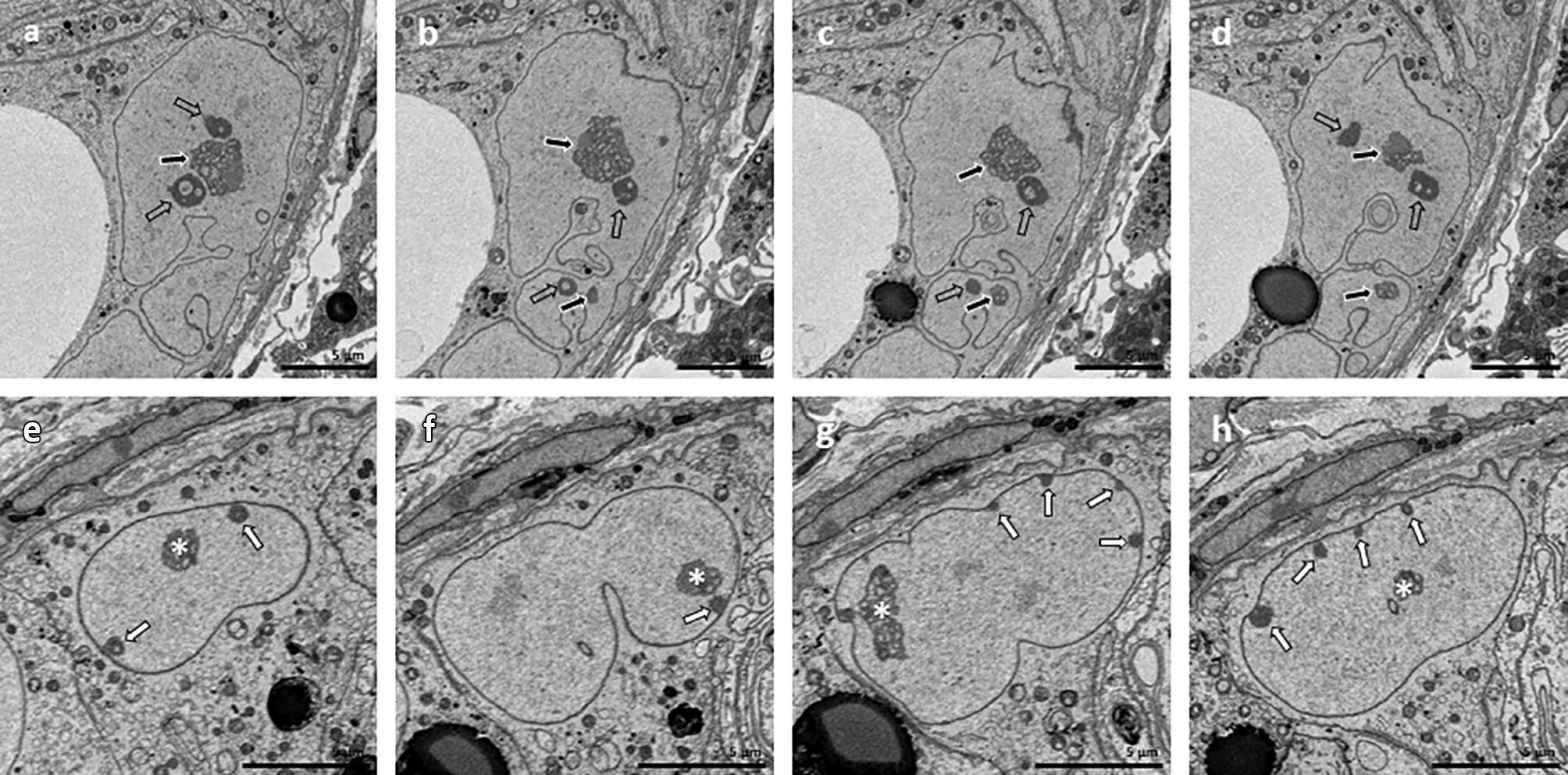
Nuclear morphology of two SCCx43KO^−/−^ Sertoli cell nuclei (SCN) imaged via serial block-face scanning electron microscopy. The figure shows different consecutive images of two SCN in each row (slice numbers: **a** 525, **b** 541, **c** 545, **d** 551, **e** 485, **f** 512, **g** 543, **h** 562). In the top row, the nuclear envelope is numerously and deeply indented, particularly at the lateral and basal surface (**a**–**d**). Two nucleoli (**a**–**d**, black arrows) and several satellite chromocenters (**a**–**d**, transparent arrows) can be found. It seems that two SCN are connected via a small bridge (**a**,**b**,**d**), possibly representing a SCN in the late telophase of mitosis. In the bottom row, the SCN shows several heterochromatin patches along the nuclear envelope (**e**–**h**, white arrows). One deep indentation of the nuclear envelope is visible in the SCN ‘s centre (**f**). The other clefts are more shallow (**f**,**g**). Nucleolus-like structures appear at different locations within the SCN (**e**–**h**, white asterisks). Scale bars = 5 µm.

In addition, in one of the SCCx43KO^−/−^ mice, two basally located SCN showing a special phenotype, different from the other SCN, were detected. The first one appeared like two SCN, which were connected via a small nucleoplasmic bridge, forming one distinctly larger SCN, than the others of the investigated tubular section (Fig. 5a–d). Each of these connected SCN owned a nucleolus, one of which was smaller and associated with the smaller nucleus. While the smaller SCN contained only one satellite chromocenter, the larger one contained six of these. In total, the SCN showed multiple and deep indentations of the nuclear envelope, particularly at the lateral and basal surface. Taken together, the described morphological features indicated that these two SCN were probably nuclei of proliferating SCs.

The second SCN showed mostly shallow indentations of the nuclear envelope (Fig. 5e,g,h). One deep indentation could be found in the SCN’s centre (Fig. 5f). Several small heterochromatin patches were distributed along the nuclear envelope. Most obvious are the convoluted, euchromatic nucleolus-like structures at different locations within the SCN.

**Figure 6:**
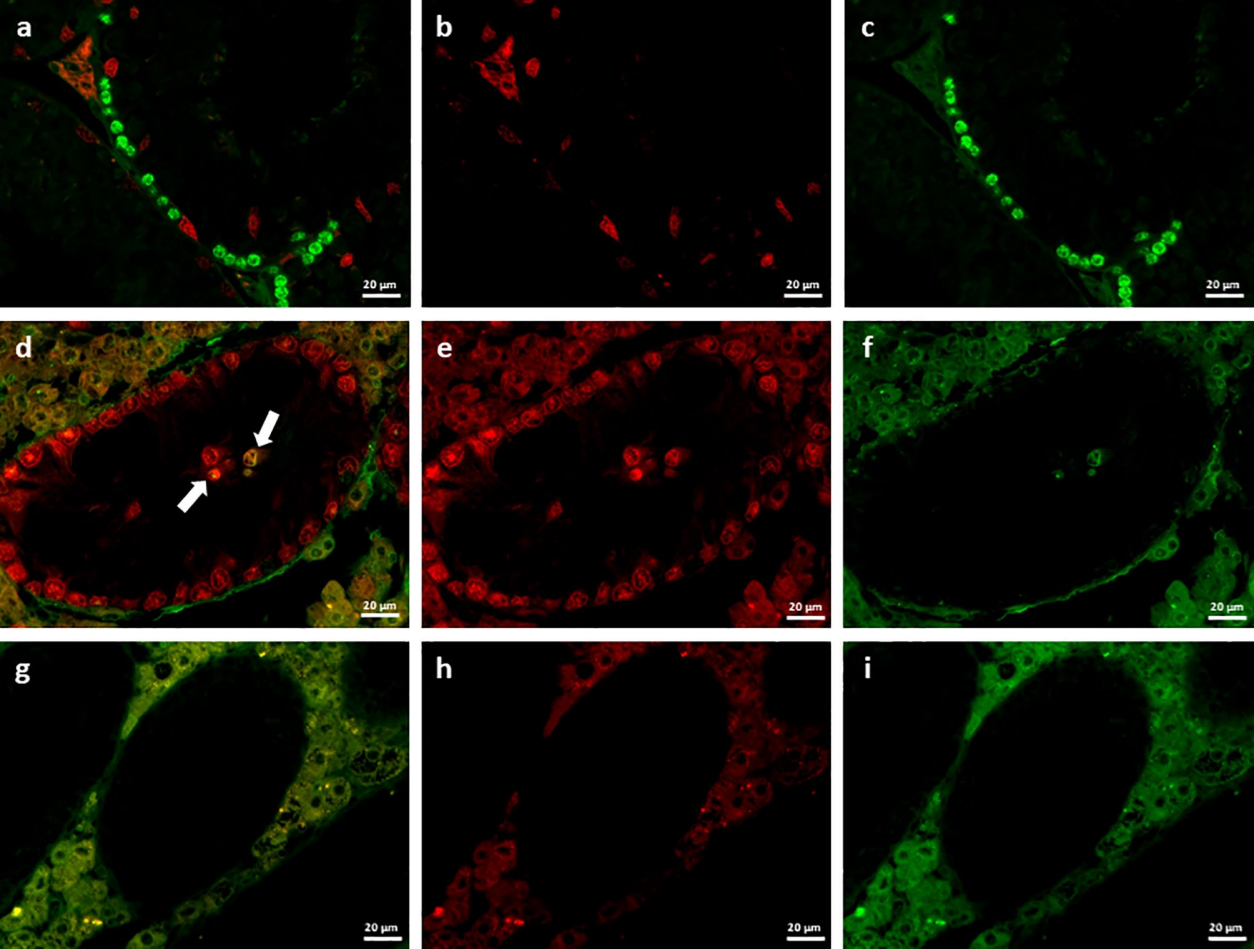
Double immunofluorescence for Sox9 and BrdU. Seminiferous tubules of adult wild type (WT) (**a**–**c**) and SCCx43KO^−/−^ (**d**–**i**) mice. All tubules contain Sox9 immunopositive Sertoli cell nuclei (SCN) (red). In SCCx43KO^−/−^ mice SCN of Sertoli cell (SC)-clusters stain also positive for Sox9, emphasizing that clusters are composed of SCs. BrdU positive germ cells occur at the tubules basal compartment of adult WT mice (**a**,**c**, green), whereas in adult mutants only single tubules contain a few of them (data not shown). Interestingly, clustered SCs in SCCx43KO^−/−^ mice occasionally show a nuclear double staining for both Sox9 and BrdU (**d**, white arrows, yellow), indicating that these cells maintain their proliferative potential. Negative control (**g**–**i**). Scale bars 20 µm.

In order to determine the proliferation status and thus maturation state of SCs in adult mutants more precisely, double-immunofluorescence staining for Sox9 and BrdU was performed (Fig. 6a–i). Single clustered SCs were immunopositive for both Sox9 and BrdU (Fig. 6d, white arrows), indicating that these cells are proliferating SCs. This phenomenon could so far only be observed in clustered SCs, whereas basally located SCs stained immunonegative for BrdU. However, not every SC-cluster contained BrdU immunopositive SCs (data not shown).

## Discussion

Our results highlight that Cx43 plays a crucial role not only for tubular morphology, but also for SC ultrastructure and maturation. Germ cells are embedded in a stage-specific manner between the long cytoplasmic branches of SCs, which extend to the tubular lumen^2,26^. In the present study, seminiferous tubules of SCCx43KO^−/−^ mice were vacuolated and the tubular lumen was filled with entangled SC processes. Since there was no evidence of lipids in tubular vacuoles, the vacuolated phenotype occurs from entangled SC processes, occluding the tubular lumen. The question arises if the convoluted apical SC surface is a result of germ cell absence or a direct effect of SC-specific Cx43 loss. The absence of structural support of differentiated germ cells in SCCx43KO^−/−^ mice might cause these long disorganized cytoplasmic processes. The fact that various other studies, using different methods and mouse models, resulting likewise in a SC-only phenotype, display a comparable tubular morphology, strengthens the latter hypothesis^29–33^. Following experimental cryptorchism, seminiferous tubules of rats developed vacuoles between neighbouring SCs at the basal compartment of seminiferous tubules. With persistent cryptorchid state, these vacuoles were no longer visible but parallel arranged smooth membrane stacks appeared, which were assumed to be a result of vacuole disintegration^34^. We found similar vacuoles and membrane-like stacks in SCCx43KO^−/−^ mice, which are presumably a consequence of permanent germ cell absence in the adluminal compartment of the seminiferous epithelium. Since the almost complete lack of apical germ cell-SC-interactions in mutants, SCs showed a decrease in height and seemed to have collapsed.

SCs are considered to be polarized cells, with a nucleus residing near the basement membrane, except at the time of spermiation^3^. Seminiferous tubules of SCCx43KO^−/−^ mice contained intratubular SC-clusters. SCN of these clusters were distinctly located away from the tubular wall, indicating that these cells were either removed from the basal compartment of the seminiferous epithelium as Sridharan et al. suspected^18^ or merely lost their cellular polarity. Since the basement membrane contact is thought to be essential for SC survival^35^, it is more likely that clustered SCs lost their polar phenotype. Senescence appears to have influence on SC polarity, since SCN in ageing rats lose their closeness to the basement membrane without inducing cellular death^36^. Even the fact that cluster size increases with age^20^, supports the theory that SC-clusters might be composed of aged non-polarized SCs. Some of these Sox9-immunopositive SCs also stained positive for BrdU, indicating proliferation as a sign for immaturity or at least an intermediate differentiation state. However, the remaining question is, what causes SC polarity loss. Previous investigations using XX-sex-reversed mice, showing similar SC-clusters also with morphological features of immaturity, hold the absence of germ cells accountable for clustered SCs^33^. Since differentiated germ cells are also absent in SCCx43KO^−/−^ mice, they could be a missing landmark for SC polarity.

Moreover, changes in lipid metabolism are considered as another age-related process^37^. In the present study, only few Sudan III-stained granules occurred in SCs of WT mice. Surprisingly, an accumulation of lipid droplets was found in intratubular SC-clusters of same-aged SCCx43KO^−/−^ littermates. Degenerating spermatogenic cells and residual bodies are degraded and digested by SCs through phagocytosis^3,38,39^, forming lipid inclusions. The number and size of lipid droplets in SCs were shown to be stage-dependent, increasing in number during the second half of the seminiferous cycle^39–41^. These lipids are predominantly degraded via the β-oxidation pathway, providing an important source for SC-ATP synthesis^42^. Since germ cell numbers are already reduced in neonatal SCCx43KO^−/−^ mice plus few differentiating spermatogonia and early spermatocytes are solely seen in pubertal mice^22^, it is unlikely that lipid droplets occur in SCs of adult mutants due to directly preceding apoptosis of differentiated germ cells. Moreover, no enhanced apoptosis was apparent. Therefore, the increased number of lipid inclusions in SCs of SCCx43KO^−/−^ mice might have another cause. Two possible sources of augmented lipids in clustered SCs are conceivable: (1) an enhanced energy need resulting in an increased accumulation of intracellular lipids as a substrate for ATP synthesis, or (2) a decreased lipid degradation. Since germ cells are mostly absent or seminiferous tubules of mutants display a SCO phenotype, there is a lack of phagocytozeable cells. The question arises, where these lipids come from. Nevertheless, since clustered SCs show signs of enduring proliferation, the former hypothesis of enhanced energy need seems likely. Alternatively, Cx43-loss might directly affect the lipid metabolism in SCs, but further studies are needed to investigate the potentially altered pathways. Nistal et al., who examined the SC morphology in human testes showing SCO syndrome, also found abundant lipid droplets in SCs with deeply infolded nuclei and suggested accelerated SC ageing as a cause for this phenotype^43^. Nevertheless, since clustered SCs of mutants show signs of enduring proliferation, which is a feature of immaturity, it is unlikely that these cells are aged. Besides seminiferous tubules, the testicular interstitium also demonstrated alterations. Lipid droplets were more abundant in the interstitium of mutants compared to WT littermates. Since Noelke et al. confirmed a LC hyperplasia in SCCx43KO^−/−^ mice^44^, increased numbers of interstitial lipid droplets could be considered as confirmation that SC-specific Cx43 loss also has an impact on LCs. The quantification of lipid droplets in SCs resulted in a notably higher number of lipid droplets in mutant mice. This difference was even more prominent in the older mutant. Since it is known that the number of clustered SCs increases with age^18,20^, this could be a possible reason for these findings. To exclude SC-clusters as the sole cause for higher numbers of lipid droplets in SCCx43KO^−/−^ mice, the number of lipid droplets in SCs at the basal compartment of the seminiferous tubule was determined per field of view in TEM pictures. Results show that, indeed, clustered SCs seem to contribute to a large proportion to the elevated number of lipid droplets in Cx43-deficient SCs but the number of droplets is also higher in basally located SCs.

So far, SCs are still considered as postmitotic cells, ceasing their proliferation during puberty^14–17^, but some researchers queried this opinion^45,46^. Using the present KO mouse model, it was determined that some SCs might be still proliferating in adult SCCx43KO^−/−^ mice^18,20^. From this observation arose the hypothesis that SCs are not terminally differentiated, postmitotic cells, but cells, in which proliferation is only suppressed and can be resumed under specific conditions. In our model, we proved that testes of mutants contain more SCs in comparison to WT mice^18^. Moreover, we found clustered Sox9 immunopositive SCs, which were also stained immunopositive for BrdU, and two basally located SCN with a special and interesting phenotype in one of the investigated SCCx43KO^−/−^ mice. One SCN contained several satellite chromocenters, the other one resembled a nucleus in the late telophase, both representing most likely nuclei of proliferating SCs. Since the appearance of several nucleoli is a feature of immature SCs^7,47^, it could be shown for the first time that seminiferous tubules of adult SCCx43KO^−/−^ mice do not only contain SCs with a mature or intermediate phenotype, but also immature SCs with proliferative characteristics. Even if it was found so far that basal SCs in adult mutants are immunonegative for proliferation markers and markers of immaturity^20^, it could be interesting, if these cells do express these markers anyway, since SBF-SEM revealed SCN with a proliferative or even immature phenotype. Adult SCs from the terminal segments of the seminiferous tubule are shown to be intensively proliferating and building colonies in-vitro. These cells are comparable with clustered SCN of our KO mouse model, possessing atypical nuclei with several heterochromatin patches instead of two distinct satellite chromocenters^48^.

SCs in SC-clusters demonstrate features of both aged mature and immature SCs. The fact that Cx43 is a negative regulator of SC proliferation^49,50^, emphasizes that the absent negative feedback of Cx43 might be responsible for enduring SC proliferation in SCCx43KO^−/−^ mice. By means of double-immunofluorescence for Sox9 and BrdU, we could emphasise the hypothesis of proliferating SCs in adult mutants only for clustered SCs so far, even if some basally located SCs also showed signs of immaturity and features of mitotic cells at ultrastructural level.

Unexpectedly, SCN of WT mice mainly had a round to oval shape and indentations of the nuclear envelope were rarely seen compared to SCCx43KO^−/−^ littermates. Different studies have shown that nuclear infoldings of SCs are regulated by hormones^51,52^. Nuclear clefts do also appear in other cell types and are associated with both physiological and pathological processes. For example, they occur in the dorsal root ganglion as a reaction to trauma and are postulated to be related to an enhanced nuclear metabolism^53^. Moreover, under normal conditions neurons of the neostriatum and pyramidal cells also show indented nuclei^54,55^. In the human and mammalian ovary, infoldings appear physiologically in the nuclei of granulosa cells^56–58^. Based on the theory that nuclear indentations are a sign of enhanced cellular activity and some proteins are shown to be higher concentrated within indentations^59^, SCs of mutants might be more active compared to WT mice. Nevertheless, little is known about the indentation’s functions. In different cell lines, nuclear invaginations formed channels, which were linked with the nucleolus. It was hypothesized that they work as a nucleolocytoplasmic transfer device^60^ or reduce the distance between the nucleolus and the nuclear envelope^61^. As germ cells are mostly absent in SCCx43KO^−/−^ mice, the question arises, what triggers cellular activity of SCs. Based on the fact that single clustered SCs are still proliferating in adult mutants, enduring proliferation is probably a target of enhanced cellular activity as well as augmented nucleocytoplasmic transport. The fact that heterochromatin patches are regularly detectable in SCN of SCCx43KO^−/−^ mice and that this phenotype was shown to be coherent with mitotic activity^48^, supports this hypothesis. Moreover, it was shown that nuclear envelope proteins are involved in regulation of heterochromatin formation in mammalian cells^62,63^. Since in the present study, SCN of mutants are significantly more indented than SCN of WT littermates and heterochromatin was arranged alongside the nuclear envelope, it would be interesting to find out if both alterations relate or even though they are mutually dependent.

## Conclusion

In summary, the SC-specific deletion of Cx43 leads to both structural and particularly ultrastructural alterations of tubular and SC morphology as well as metabolic changes. Moreover, the hypothesis that single SCs are still proliferating in adult SCCx43KO^−/−^ mice was underlined. The new and exciting technical approach by SBF-SEM provided the opportunity to examine the ultrastructure of a large specimen volume and to reconstruct three-dimensional nuclear morphology. Further studies are needed, to investigate potential mechanisms of metabolic alterations and functional backgrounds of morphological changes in SCs of SCCx43KO^−/−^ mice.

## Materials and methods

### Generation of SCCx43KO^−/−^ mice

Transgenic KO mice lacking Cx43 solely in SCs were generated using the Cre/loxP recombination system. All details of the breeding strategy, PCR genotyping, and confirmation of the Gja1 loss by β-galactosidase immunohistochemistry are described elsewhere^21^. Animal experiments were approved by the Animal Rights Committee at Regional Commission of Hannover, Germany (decision 33.19-42502-05-16A017, decision 33.9–42502-04-12/0877). All experiments were performed in accordance with the relevant guidelines and regulations, including ARRIVE guidelines.

### BrdU injection

For BrdU immunofluorescence, adult mice were injected with 50 mg/kg body weight BrdU (Sigma-Aldrich B5002) diluted in 10 ml/kg body weight sodium chloride solution intraperitoneally 2 h before they were sacrificed.

### Tissue sampling and treatment

Adult male SCCx43KO^−/−^ mice and WT littermates (n = 3 per genotype), aged 134–138 days, were anesthetized with carbon dioxide and sacrificed via cervical dislocation. Both gonads were immediately removed. One testis was fixed in Karnovsky’s fixative (phosphate buffered 2.0% paraformaldehyde, 2.5% glutaraldehyde fixative, pH 7.4) for SBF-SEM and TEM. The other one was either snap-frozen in liquid nitrogen and stored at − 80 °C until further processing, or fixed for 48 h in Bouin’s solution (10% formaldehyde, 4% picric acid, 5% acetic acid) followed by dissection and paraffin embedding according to standard methods. 5 µm sections were cut and stained with hematoxylin–eosin (HE) using standard protocols.

### Sudan III staining

For further analysis of intratubular vacuoles/ lipid droplets, Sudan III staining of 7 µm frozen sections was performed using standard protocols (n = 2 per genotype, aged 68 and 138 days)^65,66^. The number of lipid droplets per tubule cross-section was directly counted during light microscopy at a magnification of × 400 (n = 35 tubules per animal). For further analysis, lipid droplets were additionally quantified by ImageJ (version 1.53 k) after thresholding (n = 5 tubules per animal)^67^.

### Tissue block preparation and transmission electron microscopy

After fixation in Karnovsky’s solution, samples of WT and SCCx43KO^−/−^ mice were cut into small sections, sized 2.0 mm in diameter, followed by several washes with 0.15 M Na-cacodylate buffer (pH 7.4) at room temperature. Osmication was performed en bloc using an OTO-protocol (osmiumtetroxide, thiocarbohydrazide, osmiumtetroxide) with uranyl acetate and lead aspartate. Specimens were dehydrated in a graded series of acetone and embedded in Durcupan ACM resin (Sigma-Aldrich, St. Louis, USA). Polymerization was performed at 60 °C for at least seven days. Semi-thin sections (0.5 µm) were cut and stained with 0.1% toluidine blue dye (1 g toluidine blue diluted in 100 ml of distilled water plus 2.5 g of sodium hydrogen carbonate) to define the region of interest. Then, tissue blocks were trimmed with glass knives. Ultra-thin sections (60 nm) were cut with a diamond knife and examined in a Zeiss EM 10C transmission electron microscope (TEM) (Carl Zeiss Microscopy GmbH, Oberkochen, Germany). After that, resin blocks were mounted on an aluminium specimen pin (Gatan Pleasanton, CA, USA) with conductive epoxy glue (Chemtronics, CircuitWorks, Kennesaw, USA), followed by precise retrimming to establish the final specimen size of approximately 500 × 500 × 500 µm for SBF-SEM. After trimming, samples were sputter-coated with a gold layer. The number of lipid droplets per field of view was counted in TEM images at a magnification of × 2784.

### Serial block-face scanning electron microscopy

From each specimen block, approximately two thousand sections of 80 nm section thickness were cut in a Zeiss Merlin VP Compact SEM (Carl Zeiss Microscopy GmbH, Jena, Germany) equipped with a Gatan 3View2XP system (Gatan Inc. Pleasanton, CA, USA). Automatic imaging of the block-face was performed with a field of view of 150 × 150 µm (10,000 × 10,000 pixel, 15 nm pixel size, 0.7 µs dwell time) with 3 kV acceleration voltage in the variable pressure mode at 30 Pa.

### Image segmentation and analysis

Processing, reconstruction and analysis was performed using the freeware Microscopy Image Browser (MIB)^64^. Images from SBF-SEM were resampled (2000 × 2000 pixel, 75 nm pixel size) and converted from 16 to 8 bit format to create a smaller dataset, which facilitates further handling on the one hand and still provides sufficient resolution to analyze the structures of interest on the other hand. The contrast was adjusted to compensate for variations between the slices. For analysis, image stacks containing 500 images were aligned. Assessment criteria were nuclear shape and size, number of satellite chromocenters, heterochromatin distribution plus SC-localization within the seminiferous tubule.

### Double-immunofluorescence

Testes of adult WT and SCCx43KO^−/−^ mice were immersion fixed in Bouin’s solution for 48 h, dehydrated in 70% ethanol and embedded in paraffin. 2-µm sections were cut and mounted on glass slides (Histobond: Paul Marienfeld, Laboratory Glassware, Lauda-Königshofen, Germany). Deparaffinization was performed by several washings with xylol and tissue sections were rehydrated by immersion in a series of graded ethanols. Primary antibodies were diluted in 1% BSA for Sox9 (EMD Millipore, AB5535, 1:300) and for BrdU (Abcam, ab115874, 1:400) and incubated overnight at 4 °C in a humidified chamber. The next day, sections were incubated for 1 h with the secondary antibodies goat anti-mouse Alexa 488 (Invitrogen, 1:1000) and goat anti-rabbit Alexa 546 (Invitrogen, 1:1000) diluted in 1% BSA. Sections were dehydrated and mounted with ProLong Gold antifade reagent (Invitrogen).

## Supplementary Information

Supplementary Figure 1.

Supplementary Figure 2.

Supplementary Video 1.

Supplementary Video 2.

Supplementary Video 3.

Supplementary Information 3.
