# Supplementary figures and images for: Connexin43 represents an important regulator for Sertoli cell morphology, Sertoli cell nuclear ultrastructure, and Sertoli cell maturation
Source: Sci Rep. 2022 Jul 28;12:12898. doi: 10.1038/s41598-022-16919-4 (PMC9334284; doi:10.1038/s41598-022-16919-4)

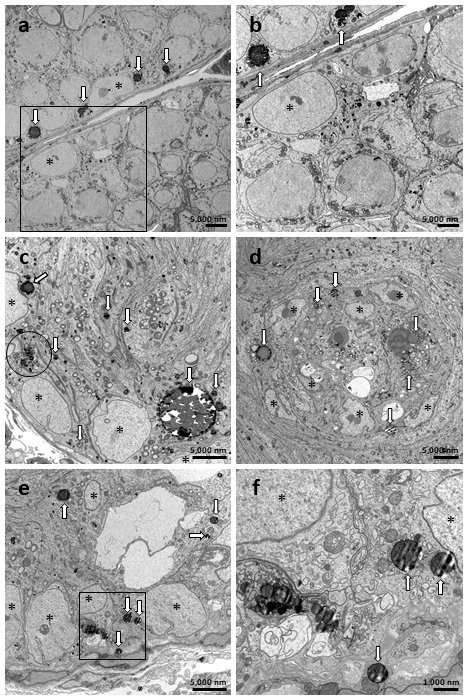

Supplement: Supplementary file 1 — Supplementary Figure 1. [file 41598_2022_16919_MOESM1_ESM.tif]

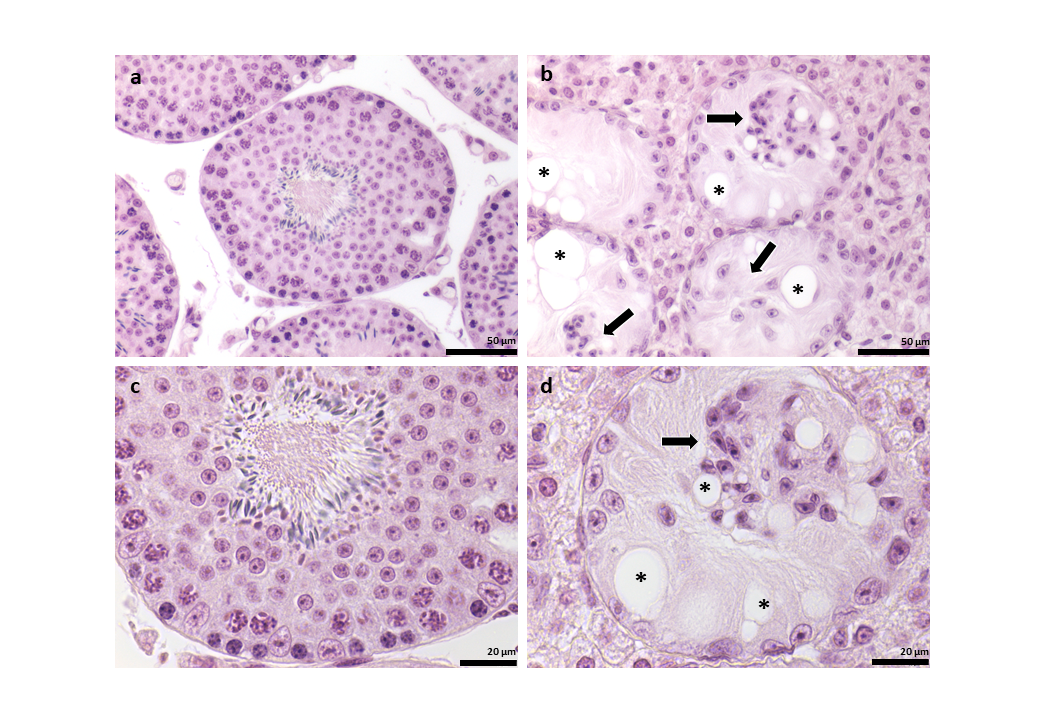

Supplement: Supplementary file 2 — Supplementary Figure 2. [file 41598_2022_16919_MOESM2_ESM.tif]
