# Supplementary material for: Connexin43 represents an important regulator for Sertoli cell morphology, Sertoli cell nuclear ultrastructure, and Sertoli cell maturation
Source: Sci Rep. 2022 Jul 28;12:12898. doi: 10.1038/s41598-022-16919-4 (PMC9334284; doi:10.1038/s41598-022-16919-4)
Supplement: Supplementary file 6 — Supplementary Information 3. [file 41598_2022_16919_MOESM6_ESM.docx]

Supplementary Figure and Video Legends

Supplementary Figure 1: TEM sections of seminiferous tubules from wild type (WT) **(a, b)** and SCCx43KO^-/-^ **(c-f)** mice. Sertoli cell (SC) nuclei are marked by black asterisks **(a-f)**. Abundant lipid droplets **(white arrows)** can be found in the SC cytoplasm of mutants **(c-f)**. In contrast to wild type mice, lipid droplets seem to be larger in SCCx43KO^-/-^ mice **(c).** Smaller lipid droplets are often arranged in groups **(c, encircled area, f shows a higher magnification of e)**. This arrangement of lipid droplets in groups can also be occasionally observed in seminiferous tubules of WT mice **(b shows a higher magnification of a)**, but the groups seem to contain less droplets compared to mutants. Scale bars **(a-e)** = 5,000 nm, **(f)** = 1,000 nm

Supplementary Figure 2: Hematoxylin-eosin-stained testes of wild type (WT) (**a**, **c**) and SCCx43KO^-/-^ (**b**, **d**) mice. Most of the seminiferous tubules of SCCx43KO^-/-^ mice are highly vacuolated (**c**, **d**, **black asterisks**) and display a Sertoli-cell-only-phenotype. Sertoli cells (SC) are either positioned near the tubules basal lamina or arranged in intratubular SC-clusters (**b**, **d**, **black arrows**). Clustered Sertoli cell nuclei (SCN) are noticeably smaller compared to basally located SCN and irregularly shaped. Scale bars (**a**, **b**) = 50µm, (**c**, **d**) = 20µm

Supplementary Video 1: Video sequence of traverse serial block-face scanning electron microscopy sections of a Sertoli cell (SC)-cluster in a SCCx43KO^-/-^ seminiferous tubule. Numerous cytoplasmic lipid droplets can be detected in mutant SCs. Polygonal shaped Sertoli cell nuclei are deeply indented with heterochromatin patches along the nuclear envelope.

Supplementary Video 2: Video sequence of traverse serial block-face scanning electron microscopy sections of a seminiferous tubule of one of the investigated SCCx43KO^-/-^ mice. Seminiferous tubules of mutants are smaller in diameter and filled with apical Sertoli cell (SC)-processes. SC-clusters can be detected in most of the seminiferous tubules.

Supplementary Video 3: Video sequence of traverse serial block-face scanning electron microscopy sections of basally located SCCx43KO^-/-^ Sertoli cell nuclei showing a mature phenotype.
